# Biomarkers and Disease Trajectories Influencing Women’s Health: Results from the UK Biobank Cohort

**DOI:** 10.1007/s43657-022-00054-1

**Published:** 2022-05-12

**Authors:** Haomin Yang, Yudi Pawitan, Fang Fang, Kamila Czene, Weimin Ye

**Affiliations:** 1 Department of Epidemiology and Health Statistics, School of Public Health and Key Laboratory of Ministry of Education for Gastrointestinal Cancer, Fujian Medical University, Xue Yuan Road 1, University Town, Fuzhou, 350122 China; 2 Department of Medical Epidemiology and Biostatistics, Karolinska Institutet, 17177 Stockholm, Sweden; 3 Institute of Environmental Medicine, Karolinska Institutet, 17177 Stockholm, Sweden

**Keywords:** Women health, Disease trajectory, Biomarkers, Phenome wide association study

## Abstract

Women’s health is important for society. Despite the known biological and sex-related factors influencing the risk of diseases among women, the network of the full spectrum of diseases in women is underexplored. This study aimed to systematically examine the women-specific temporal pattern (trajectory) of the disease network, including the role of baseline physical examination indexes, and blood and urine biomarkers. In the UK Biobank study, 502,650 participants entered the cohort from 2006 to 2010, and were followed up until 2019 to identify disease incidence via linkage to the patient registers. For those diseases with increased risk among women, conditional logistic regression models were used to estimate odds ratios (ORs), and the binomial test of direction was further used to build disease trajectories. Among 301 diseases, 82 diseases in women had ORs > 1.2 and *p* < 0.00017 when compared to men, involving mainly diseases in the endocrine, skeletal and digestive systems. Diseases with the highest ORs included breast diseases, osteoporosis, hyperthyroidism, and deformity of the toes. The biomarker and disease trajectories suggested estradiol as a risk predictor for breast cancer, while a high percentage of reticulocyte, body mass index and waist circumference were associated with an increased risk of upper-limb neuropathy. In addition, the risk of cholelithiasis was increased in women diagnosed with dyspepsia and diaphragmatic hernia. In conclusion, women are at an increased risk of endocrine, skeletal and digestive diseases. The biomarker and disease trajectories in women suggested key pathways to a range of adverse outcomes downstream, which may shed light on promising targets for early detection and prevention of these diseases.

**Supplementary Information:**

The online version contains supplementary material available at 10.1007/s43657-022-00054-1.

## Introduction

Women’s health is important for themselves, their families and the entire society. However, women experience unique healthcare challenges and are at an increased risk of several diseases, resulting in a lower quality of life (Arrospide et al. 2019). Previous studies on women’s diseases separately focused on a limited number of diseases, such as cardiovascular disease (Keteepe-Arachi and Sharma 2017), osteoporosis (Nachtigall et al. 2013), and cancer (Hemminki et al. 2012). The total spectrum of disease risks among women has not been analyzed jointly compared to men.

Despite the known biological and sex-related factors influencing the risk of diseases among women (Pucci et al. 2017), the associations between different diseases among women are still under-explored (Westergaard et al. 2019). Since cost-effective and better health care for women depends on early detection and intervention for adverse outcomes, understanding the temporal pattern of the disease network may help promote a life-course approach to women’s health and identify key indicators to mitigate the risk of future poor outcomes.

While temporal patterns of disease networks have been tested in several studies (Giannoula et al. 2018; Jensen et al. 2014; Siggaard et al. 2020; Westergaard et al. 2019), applications of this approach for exploring clustered and sequential patterns of disease networks in women are scarce. Furthermore, preclinical indicators for the diseases have not been evaluated together with the disease trajectory. As common blood and urine tests are widely performed in clinical practice during disease screening, diagnostic and surveillance procedures, these blood and urine biomarkers may be informative in the disease trajectory and suggest an earlier intervention time window for disease prevention.

This study comprehensively investigated the risk of diseases among women, compared to men, using electronic health records from the UK Biobank. We further investigated temporal patterns of the disease network among women based on pre-disease biomarkers, to identify potential indicators for the early detection of diseases.

## Materials and Methods

### Study Population

The UK Biobank is a national health project aiming to improve the prevention, diagnosis and treatment of various diseases in the United Kingdom (Hewitt et al. 2016). From 2006 to 2010, 502,650 participants were enrolled in the study across 22 assessment centers in the UK aged 40–69 years. The baseline assessment for the participants included a computer-assisted questionnaire, physical examination, and biological sample (including blood and urine) collection. Disease diagnoses were obtained from the primary diagnoses recorded in the Hospital Episode Statistics database, the Scottish Morbidity Record and the Patient Episode Database in England, Scotland and Wales, respectively, available for all participants since 1997 (Sudlow et al. 2015). Diagnoses are coded according to the International Classification of Diseases (ICD) codes. The ICD-10 system has a clear hierarchical structure for disease classification. All diseases in this study were defined based on the 3-digit ICD-10 codes (A00 to N99 and S00 to T99). Disease ascertainment ended on 31 December 2019, considering the influence of the COVID-19 pandemic.

To assess the association between female sex and disease incidence, we performed a series of nested case–control studies based on the UK Biobank data, using incidence density sampling. We first selected all the incident cases of a specific disease in the UK Biobank beyond three months after the baseline assessment and randomly sampled up to 10 participants for each case from the UK Biobank as the controls, as suggested previously (Richardson 2004) (Supplementary Fig. 1). Individual matching was performed based on age, examination center and year of participation. The controls were alive and free of the studied diseases (including diseases in the same sub-category as the studied diseases) at the date of diagnosis of the matched case (the index date). The full list of disease codes and their sub-categories can be found in Supplementary Material 1. Information on death was obtained by cross-linkage with the National Health Service (NHS) Digital or the NHS Central Register (Sudlow et al. 2015). As this study focused on incident diseases after the baseline assessment, individuals with a previous disease diagnosis before their participation were excluded when analyzing the incidence of that specific disease.

### Statistical Analysis

#### Disease Risk in Women

For each disease, we performed a conditional logistic regression model, treating female sex as the exposure and disease code as the outcome, conditioning on examination center, year of participation and year of birth. In this part of the study, we did not analyze diseases in the genital tract. As suggested by previous studies (Li et al. 2018), we only analyzed ICD codes with more than 200 incident disease cases; this resulted in 301 nested case–control studies. The odds ratio (OR) derived in a nested case–control study is theoretically largely representative of the relative risk derived from a cohort study (Goldstein and Langholz 1992). To avoid a very weak association and ensure a significant difference in relative risk between men and women (Jensen et al. 2017; Westergaard et al. 2019), diseases with ORs larger than 1.2 and *p* value less than 0.05/301(the Bonferroni corrected threshold) were considered as diseases associated with the female sex.

#### Disease Trajectory Analysis

Disease trajectory analysis was proposed to assess the strength and directionality of the association between two diseases (Jensen et al. 2014; Siggaard et al. 2020). In the first step, the association between two incident diseases (denoted by D1 and D2) was tested among women after they participated in the UK Biobank. Women with either D1 or D2 before three months after the date of participation were excluded from the analysis. As we aimed to investigate the female-specific disease trajectories, D1 and D2 should be identified as associated with the female sex in the above analysis or in the female reproductive system. We first constructed the disease pairs (D1-D2) among women. As suggested by previous studies (Han et al. 2021; Siggaard et al. 2020; Yang et al. 2019), only disease pairs with more than 50 women with these two diseases beyond three months after cohort participation were included in the analysis to ensure statistical power. The association between the two diseases was estimated using a case–control study design. We defined women with D2 as cases and randomly matched them with up to 10 participants (controls). The controls were alive and free from D2 at the time when D2 occurred in their matched cases. Cases and controls were individually matched by age at participation, year of birth, and examination center. We then tested whether D2 was associated with D1, using conditional logistic regression models, and calculated the OR to estimate the association between the disease pairs.

In the second step, for each pair of diseases identified in step 1, we used binomial tests to assess the temporal directionality (D1 → D2) of the association, as recommended by Jensen A. et al. (Jensen et al. 2014). The binomial test for D1 → D2 tested whether the probability of a patient with D1 diagnosed before D2 was significantly higher than 50% among patients diagnosed with both D1 and D2. D1 → D2 disease pairs with *p* values less than the Bonferroni-corrected threshold were then included in the next step of the analysis.

Disease pairs meeting the strength (OR > 1.2) and directionality requirement of the association test were combined into disease trajectories. To detect the subgroups of the identified disease trajectories, we further used the Louvain clustering algorithm to subdivide the network of disease trajectories into clusters. The Louvain algorithm was previously used for community identification in social network analysis, which identifies the areas of the neighbor graph to be more densely connected than the overall connectivity (Iliho and Saritha 2019).

#### Biomarkers and Disease Trajectories

To investigate the associations between preclinical biomarkers and the longitudinal patterns of multi-step disease trajectories, disease pairs meeting the directionality requirement of the association test were first combined into 3-line disease trajectories (e.g. D1 → D2 and D2 → D3 were combined into D1 → D2 → D3) with at least 10 patients passing through this trajectory to avoid chance findings. Mediation analyses were further performed to test the potential causal relationship for the 3-line trajectories treating D1 as exposure, D3 as outcome, and D2 as mediator. We used the method suggested by VanderWeele (VanderWeele 2014), which estimated the overall effect of D1 on D3, in the presence of D2, and was decomposed into direct effect, only mediation, only interaction and both mediation and interaction effects. We also estimated the percent of the total association (on the log-odds scale) between D1 and D3 that was mediated by D2. The 65 blood and urinary biomarkers and four physical examination indexes (body mass index (BMI), waist circumference, blood pressure, and heart rate) (B0) tested at the baseline in the UK Biobank (Supplementary Material 1) were analyzed to reflect the potential preclinical status of the women. As indicated by the UK Biobank, the selection of the biomarkers was based on their scientific relevance for studying a wide range of diseases.

To construct the biomarker and disease trajectory (B0 → D1 → D2 → D3) among women, the association between D1 and the biomarker was tested by considering D1 as the outcome and the biomarker (B0) from the blood or urine sample collected at the baseline as the exposure. The incidence density sampling procedure and matching criteria were the same as in the disease risk analysis. The majority of the biomarkers were standardized with a mean of zero and standard deviation of one, except for estradiol, rheumatoid factor in blood and microalbumin in urine, which were dichotomized according to their limit of detection (as more than 50% of the participants were under the limit of detection). Their associations with D1 were tested using conditional logistic regression models. All biomarkers and disease trajectories (B0 → D1 → D2 → D3) were mapped out using Cytoscape 3.5 (Shannon et al. 2003).

A flow chart of the methodology used in the analyses is illustrated in Supplementary Fig. 1. Sensitivity analyses included starting the follow-up 1 year after participating in the cohort and separating the analyses by age < 55 and age > 55.

Statistical analyses were performed using SAS (version 9.4; SAS Institute Inc, Cary, NC, USA), and R software (version 3.6.3; R Foundation for Statistical Computing, Vienna, Austria).

## Results

### Risk of Diseases in Women Compared with Men

**Fig. 1:**
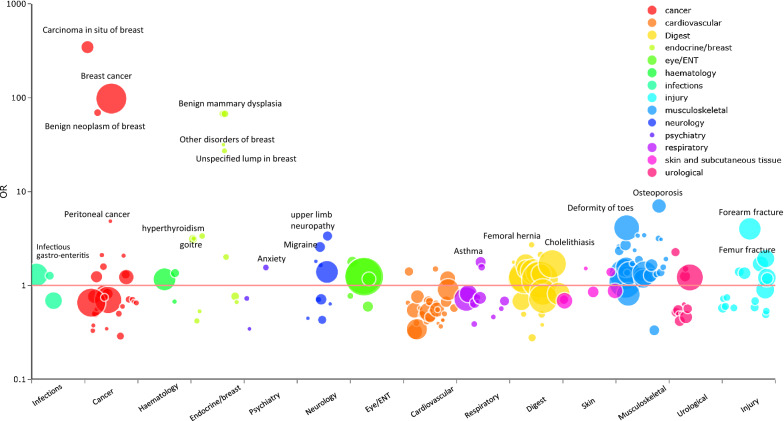
Significant odds ratios (ORs) of diseases among women, compared to men in the UK Biobank. All risk increases were statistically significant after considering the issue of multiple testing (*p* < 0.00017). The Y-axis shows the odds ratio (on the log scale) of the disease in women, compared to men. The X-axis shows the disease categories according to ICD codes. Details of the number of cases, odds ratios, and confidence intervals are listed in Supplementary Table 1

In the community-based cohort of the UK Biobank, 502,650 individuals (including 273,375 women) were included in the analysis, among whom 301 diseases were identified with more than 200 incident cases during the follow-up until the end of 2019. In the diseases analyzed, the risk of 82 diseases was higher among women than men with OR > 1.2, and *p* < 0.05/301(= 0.00017) (Supplementary Table 1, Fig. 1). Diseases with the highest ORs included breast diseases, osteoporosis, hyperthyroidism, and deformity of the toes, all of which are closely related to the hormones or the lifestyle behavior of women (Fig. 1). An increased risk of anxiety was also found in women. Sensitivity analyses focusing on diseases diagnosed beyond one year after participating in the UK Biobank cohort rendered similar results as those obtained from the main analysis (Supplementary Table 1, only one of the 82 diseases did not pass the threshold). Stratified analysis by age at the participation of the cohort showed that 83% (68 of 82) of the identified associations were consistent in young and old participants. Associations with aging-related diseases, such as cataracts, deformities of limbs and spinal and hip fractures were only found in old participants, while associations with melanocytic nevi and urinary disorders were only found in young participants.

### Disease Trajectory Network in Women

Among the 619 disease pairs tested in women from UK Biobank, 77 pairs were associated with each other and passed the directionality test (*p* value < 0.05/619 = 0.00009, Supplementary Table 2). The strongest temporal associations were found for rheumatoid arthritis (M06 → M05, OR = 221.46, 95%CI = 90.40–542.52), metastasis (C77 → C78, OR = 29.78, 95%CI = 25.15–35.26) and back pain (M54 → M47, OR = 27.31, 95%CI = 19.72–37.83).

**Fig. 2:**
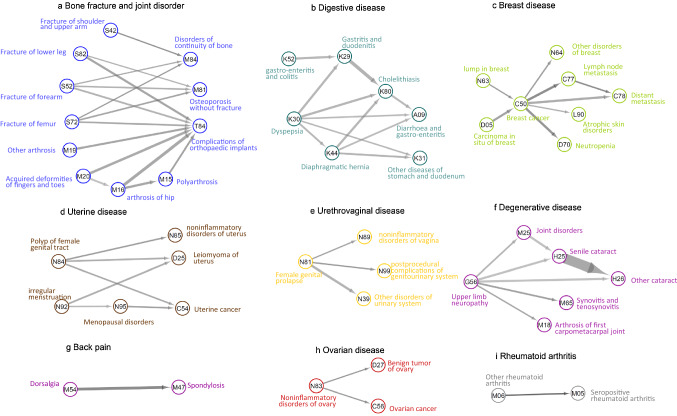
Subgroups of disease trajectory networks among women in the UK Biobank. This figure illustrates the subgroups of the disease trajectory network in our analysis. The ICD-10 codes for the diseases are shown within the circle. The width of the line connecting two circles corresponds to the number of women with this disease pair. The color of the line indicates the odds ratio of the association between the two diseases

The overall disease trajectory network is mapped in Supplementary Fig. 2, with the majority of the trajectories (more than 90%) found in both young and old women. Network clustering analysis divided the overall disease trajectory network into nine groups of diseases (Fig. 2). Among the subgroups of the disease trajectory network, the largest group included bone fracture and joint disorders, showing a trajectory from bone fracture/joint disorders to complications of internal orthopedic implants (Fig. 2a). The second subgroup consisted of digestive diseases from dyspepsia to gastroenteritis and colitis (Fig. 2b), while the third subgroup suggested the development of breast cancer from carcinoma in situ to metastasis (Fig. 2c). Other subgroups included uterine diseases, urethrovaginal disease, degenerative disease, back pain, ovarian diseases, and rheumatoid arthritis.

### Biomarkers and Disease Trajectories in Women

**Fig. 3:**
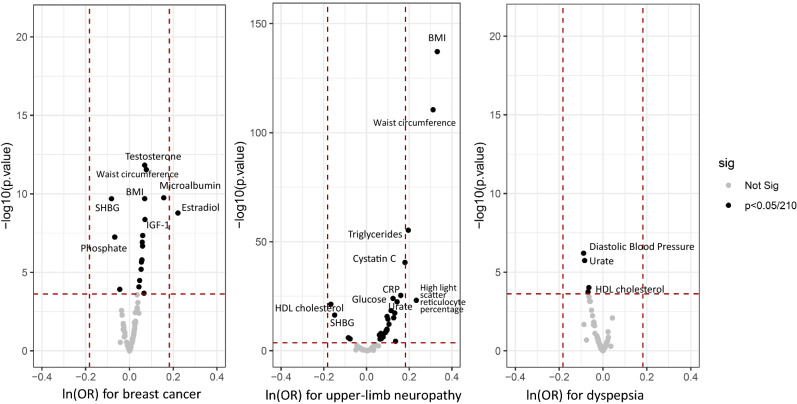
The association between biomarkers and selected diseases in the UK Biobank. This figure shows the statistically significant associations between blood and urine biomarkers and the D1s of the 3-line disease trajectories (including upper-limb neuropathy, breast cancer and dyspepsia). All the tested associations are listed in Supplementary Table 3. The horizontal dashed line indicates the *p* value < 0.05/210 (= 0.00024), while the vertical dashed line indicates an OR > 1.2 or < 0.8

**Fig. 4:**
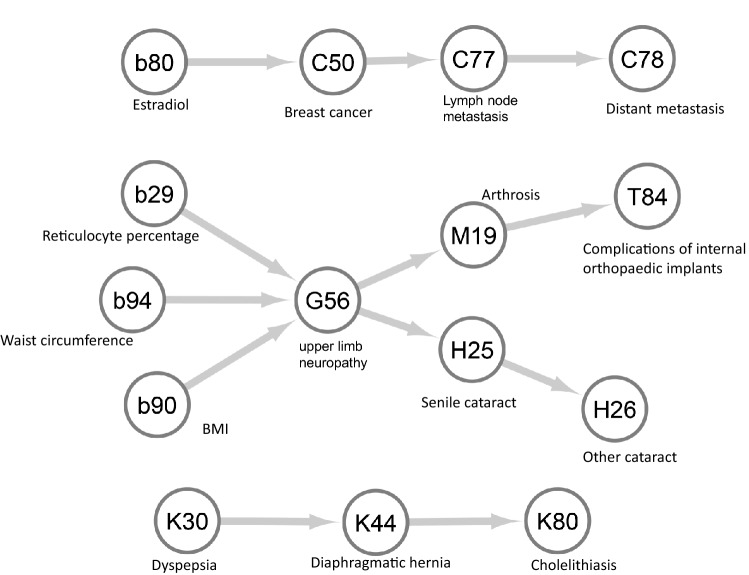
Biomarkers and disease trajectories among women in the UK Biobank. This figure shows the identified biomarkers and disease trajectories among women in the UK Biobank. For each pair of the trajectories, the codes in the circle are the ICD-10 codes for the diseases or the codes for biomarkers

Among the 77 disease pairs, four 3-line trajectories (D1 → D2 → D3) were identified with more than 10 women passing through the trajectory. In the mediation analyses, the trajectory “G56 → H25 → H26” did not pass the mediation test, and was therefore not considered as a causal relationship (Supplementary Table 3). The three distinct diseases denoted by D1 (C50 breast cancer, G56 upper-limb neuropathy, and K30 dyspepsia) in the 3-line trajectories were further tested for their associations with 65 blood and urine biomarkers and four physical examination indexes at the baseline (Supplementary Table 3, Fig. 3). We further used the identified statistically significant B0 → D1 pairs to extend the 3-line disease trajectories, resulting in the 4-line trajectories (B0 → D1 → D2 → D3, shown in Fig. 4).

In the trajectories, a higher level of baseline estradiol (OR = 1.25, 95% CI = 1.12–1.39) was associated with a higher risk of breast cancer, which further revealed the progression of breast cancer from diagnosis to lymph-node and distant metastasis. Other biomarkers such as BMI, neutrophil count, IGF-1, testosterone and sex hormone-binding globulin (SHBG) were also associated with breast cancer risk, but the ORs were below 1.2 (Supplementary Table 4).

A higher level of reticulocyte percentage was associated with a higher risk of upper-limb neuropathy (OR = 1.26, 95%CI = 1.21–1.32) in women. High BMI and waist circumference were also associated with an increased risk of upper-limb neuropathy (OR_BMI_ = 1.39, 95%CI = 1.36–1.43; OR_waist circumference_ = 1.37, 95%CI = 1.33–1.40). In addition, triglycerides, cystatin C, urate, glucose and C-reactive protein were associated with an increased risk of upper-limb neuropathy with an OR close to 1.2. The diagnosis of upper-limb neuropathy was associated with future risk of arthrosis, which might result in complications in the internal orthopedic implants. In addition, the risk of cholelithiasis was increased in women diagnosed with dyspepsia and diaphragmatic hernia.

Sensitivity analyses by starting the follow-ups one year after participation and stratifying the analyses by age groups did not render results that differ greatly from the presented results, with more than 90% of the identified associations confirmed in both age groups (Supplementary Table 4).

## Discussion

Using electronic health records in the UK Biobank, we found 82 diseases with increased risk in women as compared with men. Diseases with the largest ORs were those within the endocrine, digestive and skeletal systems. Subdivision of the female disease network revealed nine clusters of diseases, including mainly bone fracture and joint disorders, digestive diseases, and breast diseases. The biomarker and disease trajectory analysis in women showed estradiol as a risk predictor for breast cancer, while reticulocyte percentage, BMI and waist circumference were risk factors for upper-limb neuropathy and subsequent arthrosis.

In this study, we found an increased incidence of many endocrine, digestive and skeletal diseases, confirming previous knowledge (Lane 2006; Stinton et al. 2010; Wang and Crapo 1997). Many of the diseases with an increased risk in women, such as breast diseases, osteoporosis and fractures are closely related to the function of estrogen, considering the expression of estrogen receptors (ER) in breast and bone tissues (Allred et al. 2004; Khalid and Krum 2016). Similarly, estrogen may directly affect human thyroid cells through ER-dependent mechanisms, or indirectly by increasing thyroxine-binding globulin (Santin and Furlanetto 2011). Digestive diseases in the biliary system might also be influenced by estrogen as it increases the secretion of biliary cholesterol, causing cholesterol supersaturation of the bile, abdominal discomfort diagnosed as dyspepsia, and later-onset cholelithiasis (Wang et al. 2009). All these results indicate the necessity of focusing women’s health on diseases associated with estrogen for health promotion.

In addition to the hormonal effects, women are more vulnerable to coping with stressful life events and have less social support (Burani and Nelson 2020; Mandelli et al. 2015), which might explain the increased risk of anxiety observed in women in our study.

Dyspepsia was associated with the risk of diaphragmatic hernia and subsequent cholelithiasis. One of the main causes of dyspepsia is gastroesophageal reflux disease (Blair and Beltz 2006), while diaphragmatic hernia could be a consequence of long-term severe gastroesophageal reflux disease (Gordon et al. 2004). However, although the results on the association between dyspepsia/reflux and cholelithiasis are conflicting (Avidan et al. 2001; Martínez de Pancorbo et al. 1997) in previous studies, a disease trajectory from dyspepsia to cholelithiasis has been confirmed by Danish data (Siggaard et al. 2020).

In this study, the risk of breast cancer was increased in women with high estradiol. Circulating estradiol is a known risk factor for breast cancer. The association between circulating estradiol and breast cancer was supported by previous studies in premenopausal as well as post-menopausal women (Endogenous Hormones and Breast Cancer Collaborative Group et al. 2013; Fuhrman et al. 2012). We further confirmed that high circulating testosterone and low SHBG levels were associated with an increased risk of breast cancer in our study, likely due to aromatase activity in fat tissues for testosterone (de Jong et al. 2001) and the binding effect of SHBG with estrogen (Arthur et al. 2021; Missmer et al. 2004). Additionally, a Mendelian randomization study also suggested SHBG as a causal factor for breast cancer (Dimou et al. 2019). These findings argue for a close surveillance of sex hormones among women at high risk for breast cancer.

In addition to sex hormones, we also confirmed several other biomarkers to be associated with breast cancer, such as waist circumference, BMI and IGF-1. The associations between waist circumference, BMI and breast cancer were probably caused by the aromatization of testosterone to estrogens in fat tissue (de Jong et al. 2001), while women with high BMI and waist circumference have abundant fat tissue cells. The association between IGF-1 and breast cancer was confirmed by cohort and Mendelian randomization studies (Murphy et al. 2020; Tan et al. 2021), likely via biological mechanisms through mitogenic and anti-apoptotic effects (Pollak 2008).

Higher BMI and waist circumference were associated with an increased risk of upper-limb neuropathy in our study, which is consistent with previous findings for different types of upper-limb neuropathy, such as carpal tunnel syndrome and ulnar neuropathies (Rhee et al. 2021; Warner et al. 1994). This association could be explained by the extra fat tissue inside the carpal tunnel which causes hydrostatic pressure or compression of the median nerve (Becker et al. 2002).

Interestingly, our analysis also showed a strong association between a high reticulocyte percentage and an increased risk of upper-limb neuropathy in women. Historically, testing for reticulocyte percentage was only considered for patients with suspected anemia (Piva et al. 2015). Our study suggests it as an indicator for subsequent disorders of anemia, such as neuropathy and arthrosis. These associations are biologically plausible, considering the deficiency of iron, folate acid, vitamin E and vitamin B12 as causes of anemia (World Health Organization 2017). Deficiency of these nutrients could probably influence the function of the myelin sheath that surrounds and protects nerves and result in neuropathy (Staff and Windebank 2014).

The main strengths of our study include the use of a community-based cohort with abundant information on biomarkers and clinical diagnoses from electronic health records. In addition, the novel trajectory analysis provides additional information to the existing literature by clarifying the clusters and the sequential pattern of diseases with increased risk in women. Furthermore, linking the biomarkers with the disease network can assist in the exploration of key pathophysiological pathways, from preclinical biomarkers to late adverse outcomes in women. These findings, if validated by other studies, may suggest further investigation into the biological linkage among different body systems and promote the overall health of women.

We also acknowledge several limitations. The present disease network and trajectory were constructed based on electronic health records. However, the validity of electronic health records in the UK has not been thoroughly evaluated, and we, therefore, used the main diagnosis to minimize the potential bias from misclassification. Notably, although we aimed to explore sequential associations between different diseases, the disease trajectory analysis itself did not provide a substantial basis for establishing a causal relationship. The observed disease patterns might be explained by the confounding factors between diseases, including genetics, lifestyle and other environmental factors. In addition, although 80% of the biomarkers had a missing rate of less than 10%, several biomarkers had missing values because of no data return or value beyond the reportable range. We assumed the missingness as random for no data return and used complete case analysis, which might have attenuated the associations between biomarkers and diseases. Further studies are also needed to validate the identified biomarkers and disease trajectories in other settings.

## Conclusion

This study describes a comprehensive picture of diseases with increased risks among women, including mainly endocrine, skeletal and digestive disorders. The biomarker and disease trajectories in women indicate key pathophysiological pathways from biomarkers to a range of late adverse outcomes. Further investigation into these key diseases or preclinical conditions might therefore be warranted to improve women’s health.

## Supplementary Information

Below is the link to the electronic supplementary material.

Supplementary file1 (DOCX 848 kb)

Supplementary file2 (XLSX 240 kb)

## Data Availability

Data from the UK Biobank (http://www.ukbiobank.ac.uk/) are available to all researchers upon making an application. Part of this research was conducted using the UK Biobank Resource under Application 61083.
