# Supplementary material for: Biomarkers and Disease Trajectories Influencing Women’s Health: Results from the UK Biobank Cohort
Source: Phenomics. 2022 May 12;2(3):184–93. doi: 10.1007/s43657-022-00054-1 (PMC9096057; doi:10.1007/s43657-022-00054-1)

Supplementary Figure 1. Flow chart of the study population and analysis plan

1:10 case controls from UKB, matching on age, examination center and year of participation

All participants in UK biobank

(n=502,650, 2006-2019)

77 Disease pairs with increased risk of D2 after D1(Figure 2)

Disease associations

Conditional logistic regression for D2 as outcome and D1 as exposure

619 sub-cohorts of women with specific disease pairs

Binomial test for sequential directionality of disease pairs

Patient register

619 candidate disease pairs (D1-D2) : (with >50 patients )

Patient register

Build up candidate disease pairs (D1-D2) based on diseases with significantly increased risk among women and disease in female reproductive system

Diseases risk according to female sex in Figure 1

Logistic regression

Map out the biomarker and disease trajectory (Figure 4)

D1 patients in female participants as cases

1:10 female controls without D1, matched on age, examination center and year of participation

Build up the biomarker→D1→D2→D3 disease trajectory

Conditional logistic regression for D1 as outcome and biomarker as exposure

70 biomarkers examined in UK biobank

1:10 controls without D2, matched on age, examination center and year of participation, using incidence density sampling

Patients with D2 as cases

Result

Data

Method

Illustrations

Disease trajectory

Disease incidence

Patient register

**Supplementary Figure 2**. Overall disease trajectory networks in women


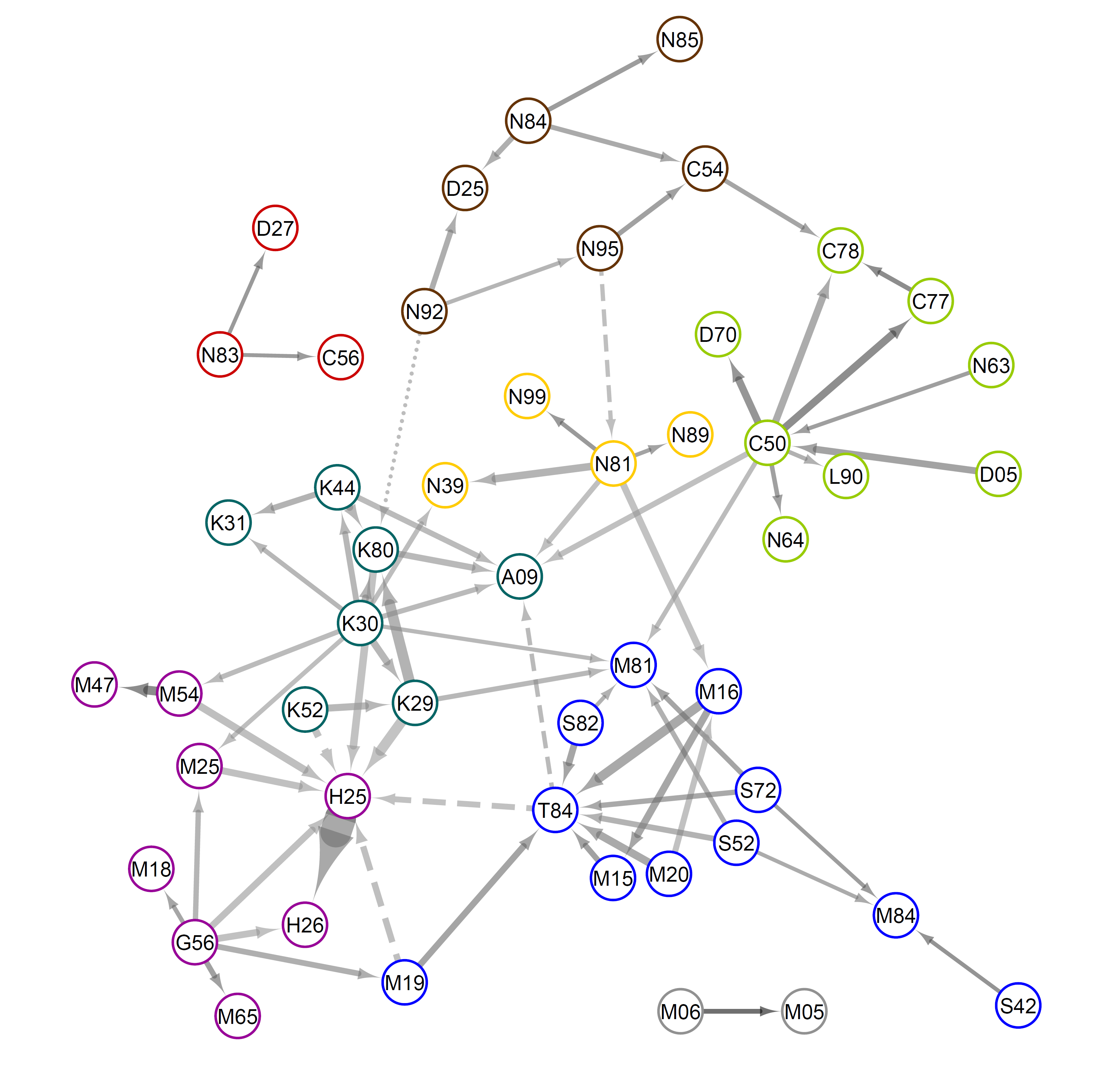

Supplement: Supplementary file 1 — Supplementary file1 (DOCX 848 kb) [file 43657_2022_54_MOESM1_ESM.docx]
